# Drug resistance profile and clonality of *Plasmodium falciparum* parasites in Cape Verde: the 2017 malaria outbreak

**DOI:** 10.1186/s12936-021-03708-z

**Published:** 2021-03-31

**Authors:** Silvania Da Veiga Leal, Daniel Ward, Susana Campino, Ernest Diez Benavente, Amy Ibrahim, Tânia Claret, Varela Isaías, Davidson Monteiro, Taane G. Clark, Luzia Gonçalves, Tomas Valdez, Maria da Luz Lima Mendonça, Henrique Silveira, Fatima Nogueira

**Affiliations:** 1 Laboratório de Entomologia Médica, Largo Do Desastre da Assistência, Instituto Nacional de Saúde Pública, Chã de Areia, Praia 719, Cape Verde; 2 Department of Infection and Biology, Faculty of Infectious and Tropical Diseases, London School of Hygiene and Tropical Medicine, London, UK; 3 Faculty of Medicine, University of Coimbra, Coimbra, Portugal; 4 Global Health and Tropical Medicine, GHTM, Instituto de Higiene E Medicina Tropical, IHMT, Universidade NOVA de Lisboa, UNL, Rua da Junqueira 100, 1349-008 Lisbon, Portugal; 5 Centro de Estatística E Aplicações da Universidade de Lisboa (CEAUL), Campo Grande, Bloco C6, Piso 4, 1749-016 Lisbon, Portugal; 6 Ministério da Saúde E da Segurança, Palácio Do Governo, nº47, Praia, Cape Verde

**Keywords:** Malaria, *Plasmodium falciparum*, Drug resistance, Genetics, Sequencing

## Abstract

**Background:**

Cape Verde is an archipelago located off the West African coast and is in a pre-elimination phase of malaria control. Since 2010, fewer than 20 *Plasmodium falciparum* malaria cases have been reported annually, except in 2017, when an outbreak in Praia before the rainy season led to 423 autochthonous cases. It is important to understand the genetic diversity of circulating *P. falciparum* to inform on drug resistance, potential transmission networks and sources of infection, including parasite importation.

**Methods:**

Enrolled subjects involved malaria patients admitted to Dr Agostinho Neto Hospital at Praia city, Santiago island, Cape Verde, between July and October 2017. Neighbours and family members of enrolled cases were assessed for the presence of anti-*P. falciparum* antibodies. Sanger sequencing and real-time PCR was used to identify SNPs in genes associated with drug resistance (e.g., *pfdhfr, pfdhps, pfmdr1, pfk13, pfcrt*), and whole genome sequencing data were generated to investigate the population structure of *P. falciparum* parasites.

**Results:**

The study analysed 190 parasite samples, 187 indigenous and 3 from imported infections. Malaria cases were distributed throughout Praia city. There were no cases of severe malaria and all patients had an adequate clinical and parasitological response after treatment. Anti-*P. falciparum* antibodies were not detected in the 137 neighbours and family members tested. No mutations were detected in *pfdhps*. The triple mutation S108N/N51I/C59R in *pfdhfr* and the chloroquine-resistant CVIET haplotype in the *pfcrt* gene were detected in almost all samples. Variations in *pfk13* were identified in only one sample (R645T, E668K). The haplotype NFD for *pfmdr1* was detected in the majority of samples (89.7%).

**Conclusions:**

Polymorphisms in *pfk13* associated with artemisinin-based combination therapy (ACT) tolerance in Southeast Asia were not detected, but the majority of the tested samples carried the *pfmdr1* haplotype NFD and anti-malarial-associated mutations in the the *pfcrt* and *pfdhfr* genes. The first whole genome sequencing (WGS) was performed for Cape Verdean parasites that showed that the samples cluster together, have a very high level of similarity and are close to other parasites populations from West Africa.

**Supplementary Information:**

The online version contains supplementary material available at 10.1186/s12936-021-03708-z.

## Background

Malaria, caused by *Plasmodium* parasites, is a global public health problem. Almost half of world population is at risk of malaria, and in 2018 there were 228 million cases and 405,000 deaths, globally [1]. In Africa, where *Plasmodium falciparum* infections dominate, 6 countries (Nigeria, Democratic Republic of the Congo, Uganda, Côte d’Ivoire, Mozambique, Niger) accounted for more than half of all malaria cases worldwide. Further, 94% of all malaria deaths occurred in the African continent. Due to malaria control activities, such as improved case management and roll-out of insecticide-treated nets and indoor residual spraying, the number of countries moving towards disease elimination has increased. In particular, the number of countries with < 100 indigenous cases increased from 17 in 2010 to 27 in 2018 [1].

Cape Verde (population size: ~ 500,000) is one of the African countries in a pre-elimination phase of malaria control, with < 1 case per 1000 population per year [2]. There were 583 indigenous cases and 5 deaths between 2010 and 2018. However, in 2017, 423 (72.6%) cases occurred in an outbreak. This year was an outlier as it corresponds to an increase of 89.9% of cases compared to previous years, and excluding it, the number of cases reported yearly since 2010 has varied between 1 and 48 (average < 20 cases) [1].

The research and studies conducted within the scope of this epidemic revealed that the probable factors associated with the increase in the number of cases were: (1) the reduction of indoor residual spraying; (2) the reduction of regular vector control interventions [3]; and, (3) ecological and environmental factors such as unusual high rainfall during 2016 [4]. In response to the 2017 outbreack, authorities have implemented measures to improve vector control interventions, which has contributed to reducing autochthonous malaria cases and mortality since 2018 [1, 4].

Despite the control efforts implemented by health authorities, autochthonous cases persist and could delay elimination targets. Malaria prevalence is unstable and autochthonous cases are restricted to the islands of Santiago (96%) and Boavista (4%), while imported cases from countries with disease transmission are recorded in all 9 islands. In recent years, local transmission has been restricted to the island of Santiago, especially in Praia city, capital of the country, where 158 cases were recorded, more than 90% of autochthonous cases from 2010 to 2016 [2]. There is typically low malaria endemicity, but there are fluctuations in morbidity depending on rainfall, with transmission normally occurring between the months of September and November [5]. However, in 2017, the malaria outbreak occurred before the rainy season, where all 423 autochthonous *P. falciparum* cases were reported in Praia city [3, 4]. Eighteen patients had at least two relapse episodes in that year, 23 further imported cases were registered [4].

All non-complicated malaria cases in Cape Verde are hospitalized and treated with artemisin-based combination therapy (ACT): artemether and lumefantrine, which targets the parasite erythrocytic asexual stage. All cases also receive the gametocytocidal primaquine drug at the start of treatment to prevent transmission and interrupt the spread of the disease. Severe cases are treated with intravenous artesunate. Levels of parasitaemia are monitored at health facilities during the period of the disease and followed up on multiple occasions, up to 42 days [6]. Success in the control and treatment of malaria depends on the clinical efficiency of ACT and avoiding drug resistance [7]. Therefore, understanding the epidemiology of drug resistance is vital for an effective drug policy, especially within the Cape Verde elimination settings [8].

The non-temporary movement of people between Cape Verde and malaria-endemic countries, particularly to and from West Africa, increases the potential for case importation, and poses a challenge to malaria elimination on the archipelago [9]. The circulation of parasites between regions also increases the risk of importing drug resistance*,* which is underpinned by mutations in the *P. falciparum* genome. For example, polymorphisms associated with resistance to several anti-malarial drugs have been identified in *P. falciparum* genes *pfdhfr* (target for anti-malaria drug pryrimethamine)*, pfdhps* (target for sulfadoxine), *pfcrt* (target for chloroquine), *pfk13* (target for artemisin) and *pfmdr1* (target for mefloquine, chloroquine). Similarly, *P. falciparum* genetics (e.g., *pfama1* gene) also gives the parasite the ability to evade the immune response of the host. Therefore, there is a need to understand the genetic diversity of circulating parasites to inform on drug resistance and transmission networks identified by finding near-identical parasite genomic sequences. To support this, epidemiological data and blood samples were collected from close contacts of patients, including neighbours and family members, thereby informing on potential risk factors for malaria susceptibility, transmission and the emergence and spread of outbreaks. Further, this study on the molecular characterization of *P. falciparum* isolates collected from patients during the 2017 outbreak will provide a baseline assessment of malaria parasite drug resistance profile and genetics in Cape Verde, from which to design population-specific diagnostics, and contribute to strengthening the country’s measures for prevention and control, in order to achieve its elimination targets.

## Methods

### Malaria patients

Malaria patients admitted to Dr Agostinho Neto Hospital at Praia city, Santiago island, Cape Verde, between July and October 2017 were enrolled. A total of 190 (from 446; 187 indigenous, 3 imported) gave informed consent to participate in the present study. The laboratory diagnosis of malaria was performed by rapid diagnostic tests and microscopy.

All 190 patients that agreed to participate remained hospitalized for at least 3 days and were discharged from hospital only after laboratory confirmation of *Plasmodium *spp*.* negative blood smear and clinical evaluation; follow-up assessments were performed on days 7, 14, 21, 28, 35, and 42 post-start of treatment. The management of participating patients strictly followed the guidelines from the Ministry of Health [6].

### Data and sample collection

Data were obtained from laboratory analysis and from a questionnaire. Of the 190 malaria cases enrolled in this study, 131 (68.9%) cases and their neighbours and family members (n = 137; without malaria symptoms), answered a questionnaire in a community setting. This questionnaire was designed in both Portuguese and Crioulo, which captured sociodemographic data, present and past about malaria and fever, travel information and signs and symptoms consistent with malaria. Species identification and parasite quantification was performed by microscopic observations of thick and thin blood smears. Blood drops of malaria cases and their neighbours/family obtained by finger prick were collected on filter paper and stored at − 20 °C for later molecular characterization of parasites and antibody (AMA1 and MSP1) detection. Parasite DNA was extracted using Chelex [10] and species confirmation by PCR [11].

### AMA1 and MSP1-19 indirect ELISA

Dried blood spot elution was performed as previously described [12]. In brief, 137 blood spots from samples collected in 2017 were equilibrated to ambient room temperature before opening, and 2.5-mm diameter discs were cut from Whatman 3 MM paper using a leather punch. Each disc was then eluted in a 96-deep-well plate containing 150 μl PBS/0.05% (v/v) Tween 20/0.05% (w/v) sodium azide and incubated at ambient room temperature for > 18 h with gentle rotary agitation ~ 100 rpm. Serum was diluted to a 1:100 dilution. IgG antibodies were detected by indirect ELISA using MSP1-19 (Wellcome Genotype) and AMA1 (3D7) antigens expressed in *Escherichia coli* strain BL21, as described by Okech et al*.* [13]*,* and by Hoder [14]. AMA1 and MSP1-19 antigens were coated onto 96-well ELISA plates (Immulon 4HBX, Thermo) with coating buffer (carbonate-bicarbonate) in a 50-µl volume at a concentration of 0.5 µg/m and incubated a 40 °C overnight. Plates were washed several times using PBS plus 0.05% Tween 20 (PBS/T) and blocked with 1% (w/v) skimmed milk powder in PBS/T (block solution). The previously eluted serum was used at 1:400 working dilution into block solution. European confirmed negative and pooled hyperimmune positive serum were used as negative/positive control samples. Serum was incubated at 40˚C overnight and washed in PBS/T. Rabbit anti-human HRP conjugated secondary antibody (Dako) was used at a concentration of 1:15,000 in PSB/T. TMB substrate was added followed by a 15-min room temperature (RT) incubation. H_2_SO_4_ stop solution was added, followed by plate reading at 450 nm.

### Candidate gene sequencing and analysis

A total of 38 samples with the highest parasitaemia (2000–274,000 parasites/μl) were selected for sequencing of anti-malarial drug resistance loci, including *pfmdr1* (PF3D7_0523000; including codons 86, 184 and 1246), *pfk13* (PF3D7_1343700; including codons 417 to codon 714), *pfdhfr* (PF3D7_0417200.1; including codons 50, 51, 59, 108 and 164) and *pfdhps* (PF3D7_0810800.1; including codons 431, 436, 437, 540). Genes were PCR amplified and products cleaned using SureClean (Bioline, USA) following manufacturer instructions. The PCR products were analysed by electrophoresis on a 1.5% agarose gel stained with GreenSafe Premium (Nzytech) to confirm amplification. The primer pairs and thermocycling conditions are summarized in Additional file 1: Table S1. The PCR products were sequenced using Sanger capillary platform, and the resulting sequences were analysed using Geneious software (v4.8.5) and the laboratory-adapted strain 3D7 was used as reference. A fragment of the *pfcrt* gene (PF3D7_0709000; including codons 72, 74, 75, 76) was also examined. Three dual-labelled probes with sequences complementary to each of the *pfcrt* 72–76 haplotypes (denoted as CVIET, CVMNK, SVMNT), were included in the real-time PCR reaction, each containing a different fluorescent molecule [15, 16]. Two amplicons (479 and a 516 bp) of the gene *pfama1* (PF3D7_1133400) were amplified in 20 samples from 2017 and 4 samples from the 2016, using protocols published elsewhere [17]. Amplicons were amplified with primer pairs and thermocycling conditions summarized in Additional file 1: Table S1. PCR products were analysed by electrophoresis on a 1.5% agarose gel stained with ethidium bromide and sequencing using the same methodology described above.

### Selective whole genome amplification and whole genome sequencing

Seven samples from 2017 with the highest parasitaemias (3141–126,690 parasites/μl) and meeting whole genome sequencing (WGS)-required DNA quality were selected for WGS, together with 4 samples from 2016. Genomic DNA was extracted from dried blood spots using the QIAamp DNA Investigator Kit (QIAGEN, Germany) as per manufacturer’s directions. DNA concentrations were measured using the Invitrogen Qubit Fluorometer. A selective whole genome amplification (SWGA) strategy was applied prior to WGS, following previously published protocol [18]. Oligonucleotides that preferentially bind with high frequency to the *P. falciparum* DNA, and rarely bind to the human DNA, were used for SWGA. All SWGA reactions were carried out in a UV Cabinet for PCR Operations (UV-B-AR, Grant-Bio) to eliminate potential contamination. Briefly, a maximum of 60 ng of gDNA (minimum of 5 ng) was added to a total 50 µl reaction alongside with 1X phi-29 buffer (New England Biolabs), 1X bovine serum albumin, 1 mM dNTPs, 2.5 µM of primer mix, 30 U phi-29 DNA polymerase (New England Biolabs) and water. The reaction was carried out on a thermocycler with the following step-down programme: 5 min at 35 °C, 10 min at 34 °C, 15 min at 33 °C, 20 min at 32 °C, 25 min at 31 °C, 16 h at 30 °C, and 10 min at 65 °C. The 5 samples, selected for SWGA, were then whole genome sequenced on a MiSeq (Illumina). The QIAseq FX DNA Library Kit (QIAGEN) was used for library preparation according to the manufacturer’s protocol, with a 20-min fragmentation step. Library DNA concentration was analysed using a Qubit 2.0 fluorometer. All sequencing reactions were performed using 2 × 150 bp reads.

### Whole genome sequence data analysis

Raw fastq files obtained after the MiSeq run were trimmed using *trimmomatic* set to default parameters [19], and aligned to the *P. falciparum 3D7* reference genome (PlasmoDB) using *bwa-mem* software [20]. SNPs were identified using *samtools* software [21] and filtered for quality based on previously described methods [22, 23]. The coverage of each nucleotide was analysed using *sambamba* [24], which was set to include SNPs with only a coverage of fivefold or above. To investigate the population structure of *P. falciparum* parasites, a distance matrix was created which was based on a matrix of pair-wise identity calculated from the SNPs present in each sample. Using the distance matrix, a neighbourhood-joining tree was produced and visualized in iTOL [25]. WGS data from an extra 400 publicly available samples from Africa, South America, South Asia and Southeast Asia were also used for analysis [22, 23, 26]. A neighbourhood-joining tree was used to investigate the clustering of samples from West Africa.

For genetic distance comparisons between samples, a clustering approach using a Manhattan distance matrix of pair-wise identity by state values was calculated from the sub-set of SNPs available for each pair-wise comparison using the R software.

### Statistical analysis

Regarding data collected by questionnaire (among other sociodemographic data, present and past about malaria and fever, travel information and signs and symptoms consistent with malaria), two groups were compared: malaria cases (n = 131) and neighbours/family members (n = 137).

Differences in categorical variables were tested by Chi-Square or Fisher exact tests. Differences in continuous variables were assessed using t-tests (after checking for normality and the homogeneity of variances), or alternatively, Mann–Whitney-Wilcoxon non-parametric test. Malaria cases and neighbours and family were analysed following an unmatched design, this decision was also supported by recent discussion about the efficiency of matched and unmatched studies [27, 28], balancing sample size and efficiency. Thus, unconditional multiple logistic regression models were used to to explore simultaneously the association of different independent variables with the binary dependent variable (malaria case *vs* close contacts), controlling for potential confounding. Multiple models included all variables with p < 0.20 in the simple logistic regression models. Adjusted odds ratios (OR) were presented with 95% confidence intervals (95% CI).

## Results

### Characterization of malaria cases

From the 446 cases in the 2017 outbreak, 190 blood samples collected from malaria patients before treatment administration, including 187 indigenous and 3 imported infections, were analysed. All samples were *P. falciparum* positive (PCR confirmed) and one patient presented with a mixed *P. falciparum* and *Plasmodium malariae* infection, undetected by microscopy. Considering indigenous cases group, only 27 (14.4%) were children (≤ 16 years), and the majority of participants were adults (n = 160, 86.6%; range: 17–91 years old). All imported cases were young adults, 2 were 33 years and one 31 years of age.

**Fig. 1:**
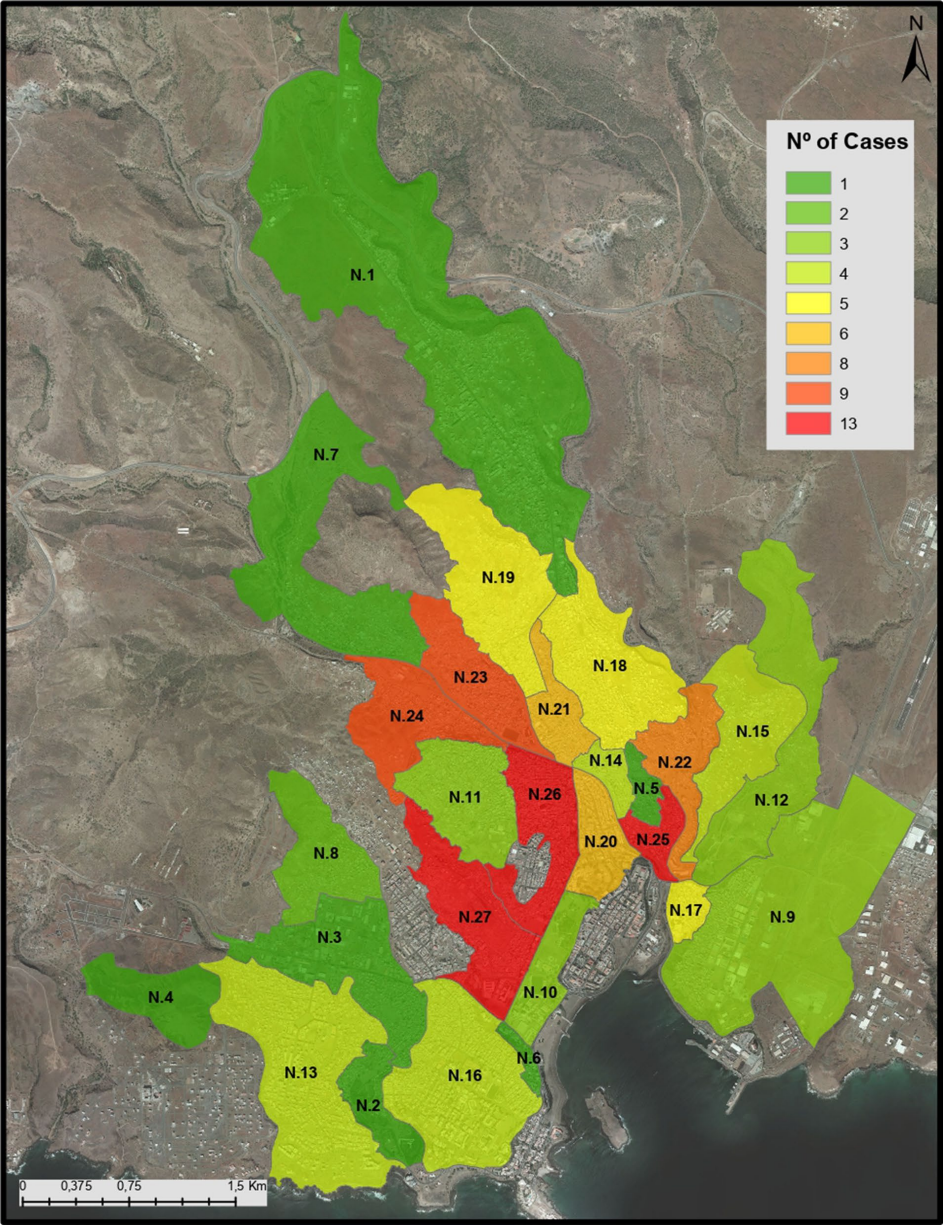
Number of malaria cases in Praia city per neighbourhood (N). N.1—São Filipe; N.2—Fonton; N.3—Tira Chapéu; N.4—Monte Vermelho; N.5—Achadinha Pires; N.6—Chã de Areia; N.7—São Pedro Latada; N.8—Bela Vista; N.27—Várzea; N.9—Achada Grande Frente; N.10—Tahiti Chã de Areia; N.11—Achada Eugénio Lima; N.12—Jamaica/Agua Funda; N.13—Palmarejo; N.14—Lém Cachorro; N.15—Achada Mato; N.16—Achada Santo António; N.17—Lém Ferreira; N.18—Ponta de Agua; N.19—Safende; N.20—Fazenda Sucupira; N.21—Vila Nova; N.22—Coqueiro Castelão; N.23—Calabaceira; N.24—Pensamento. N.25—Paiol; N.26—Achadinha

Malaria cases were distributed throughout Praia city (Fig. 1) and consistent with official statistics. Varzea, Paiol and Achadinha were the neighbourhoods with highest number of cases (n = 13) (Fig. 1). There were no cases of severe malaria, nonetheless 18 patients presented with hyperparasitaemia (> 100,000 parasites/µl blood) ranging from 105,840 to 400,000 parasites/µl blood. One fatality was registered, but malaria was not the cause of death. The 3 most common self-reported symptoms in malaria cases were fever, headache and chills. At day 3 of hospital treatment, Giemsa-stained smears were negative for *Plasmodium spp.* for all patients. The length of hospital stay of malaria cases varied between 3 days to 1 month, with a median of 4 days (Table 1). None of the patients evidenced signs of severe malaria. Extended hospitalizations were clinically justified by other medical conditions.

**Table 1:** Characteristics of the malaria patients and their close contacts (family and neighbours)

Variables n (%)	Malaria cases (n = 131)	Close contacts (n = 137)	*p*
Age in years (Mean ± SD)	32.4 ± 15.3	40.3 ± 18.6	< 0.001
Median (Min–Max)	30 (3–86)	39 (1–83)	
Gender n (%)			< 0.001
Male	86 (65.6)	37 (27.0)	
Female	45 (34.4)	100 (73.0)	
Marital status n (%)			< 0.001
Single	96 (73.3)	66 (48.9)	
Married	12 (9.2)	25 (18.5)	
Civil union	23 (17.6)	38 (28.1)	
Divorced/widowed	0 (0.0)	6 (4.4)	
Nationality n (%)			1.000
Cape Verde	130 (99.2)	135 (98.5)	
Other	1 (0.8)	2 (1.5)	
Professional status n (%)			0.150
Unemployed	18 (13.8)	18 (13.4)	
Primary sector	1 (0.8)	0 (0.0)	
Secondary sector	5 (3.8)	11 (8.2)	
Tertiary sector	74 (56.9)	79 (59.0)	
Retired	1 (0.8)	5 (3.7)	
Student	31 (23.8)	21 (15.7)	
History of malaria n (%)			1.000
Yes	2 (1.2)	3 (2.2)	
No	129 (98.5)	133 (97.8)	
Length of hospital stay in days		–	
Median (P_25_-P_75_)	4 (3–4)		
(Min–Max)	(3–30)*		

^*^ Hospital stay was extended in some patients, based in individual clinical evaluation

### Sociodemographic and epidemiological characterization

Table 1 (and Additional file 1: Table S1) shows sociodemographic and epidemiological information of a set of 131 patients and 137 close contacts (family members or neighbours, without malaria symptoms). Almost all participants (265/268; 98.9%) were Cape Verde nationals. Malaria cases were predominantly single (73.3%), male (65.6%) and younger than non-malaria cases (all p < 0.001). Malaria cases tended to travel less in the previous 6 months (5.6%) than their close contacts (11.8%), but this difference was marginally non-significant (p = 0.060). Students and tertiary-sector activities accounted for 80.2% malaria cases and 73.0% non-malaria group. Geographical locations of the cases suggested (Fig. 1) some clusters in Varzea, Paiol, Achadinha, Calabaceira, and Pensamento, corresponding to 43.5% of all cases.

Multiple logistic regression models suggested increased risk of malaria from being male (OR = 4.99; 95% CI 2.90–8.58) and single (OR = 1.92, 95% CI 1.02–3.58), with potentially lower risk from higher age (OR = 0.98; 95% CI 0.97–1.00). All malaria cases were PCR positive for *P. falciparum,* while all close contacts (n = 137) were negative. Serologic detection of *P. falciparum* anti-AMA1 and MSP-1-19 IgG/IgM was also negative for all 137 neighbours and family members.

### Drug resistance profile of malaria cases

A sub-set of the malaria cases (n = 38/190) was investigated for the presence of alleles associated with drug resistance. The chloroquine-resistant CVIET haplotype (*pfcrt* gene) was detected in all samples analysed except one that carried CVMNK (wild type haplotype). No mutations in the *pfk13* gene, associated with artemisinin resistance, were identified. All *pfk13* sequences analysed were identical to that of 3D7 reference strain except one that carried two mutations R645T and E668K, which have not been observed in other isolates [22]. Sulfadoxine-pyrimethamine (SP) drug-resistant associated alleles were also surveyed. For the *pfdhps* gene the wild-type haplotype (S436/A437/K540) was present in all samples analysed. On the other hand, for *pfdhfr* the triple mutation S108N/N51I/C59R was detected in all analysed samples (n = 12). The codons 86, 184 and 1246 from the *pfmdr1* gene were also analysed. The majority of samples (89.7%) carried the haplotype NFD (meaning N86/F184/D1246) that is associated with artemether + lumefantrine tolerance and the others were wild-type for the 3 codons (N86/Y184/D1246). No other polymorphisms were detected for the samples and regions analysed in the 5 genes.

### Population genetic analysis

WGS was performed in 7 autochthonous samples with the highest parasitaemia and 4 samples from 2016. The resulting data were compared to publicly available *P. falciparum* global genomic sequences [23, 26], in order to explore their genetic diversity. Prior to WGS, SWGA was used to increase the amount of parasite DNA. The success of the SWGA is very dependent on parasitaemia and the quality of the DNA. After WGS, the samples from 2016 were excluded for analysis due to having less than 1% of genome sequenced. The WGS data confirmed the drug resistance profile results, with all samples having the *pfcrt* CVIET haplotype, the Y184F mutation in *pfmdr1* gene, the triple mutation S108N/N51I/C59R in *pfdhfr* and wild type alleles for *pfdhps*.

**Fig. 2:**
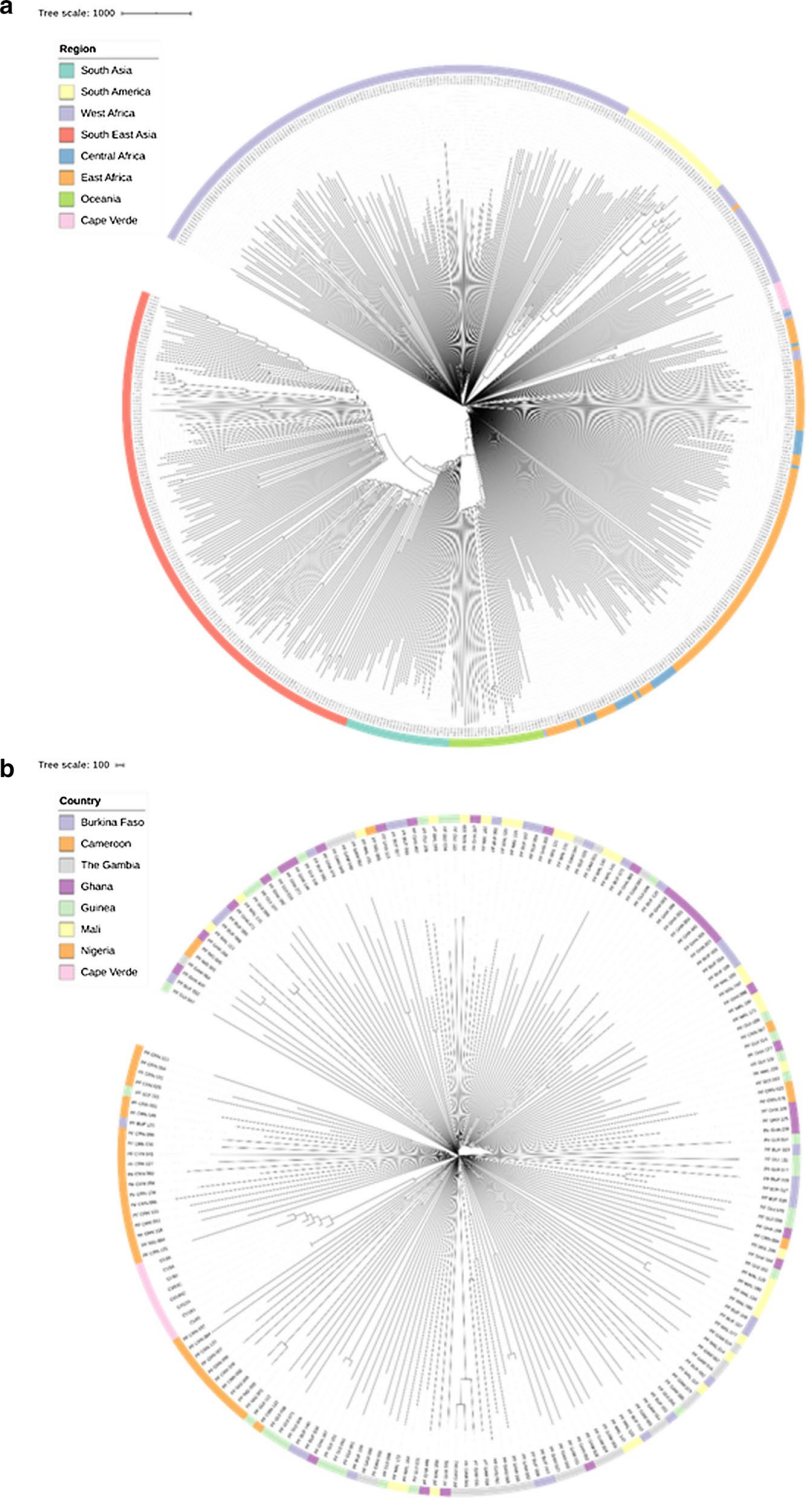
Neighbour-joining trees constructed using SNP data from Cape Verde samples using global samples. Cape Verde (pink) clusters together with other samples from Africa (**a**) particularly West Africa (**b**)

A neighbour-joining tree was constructed using SNP data and demonstrates that the Cape Verdean samples group together, and cluster with samples from West African (Fig. 2). To investigate the genetic similarity between samples, an analysis of SNP sharing was performed, including samples from other locations in Africa and Asia. The samples from Cape Verde shared > 99% of SNPs, while when compared with other samples on average 97% (maximum 97.6%) of SNPs were shared, a value normally observed between samples from different regions (e.g., African and Asian samples share on average 96.6% SNPs). To further explore the genetic diversity, two fragments of the *PfAMA1* gene were sequenced for 20 samples, including the samples from 2016. The results show that all amplified samples that were successfully sequenced (n = 18 for 2017; n = 3 from 2006) share the same haplotype in both amplicons.

## Discussion

### Insights into the epidemiology of Cape Verde malaria

There is a lack of studies into the genetics and epidemiology of malaria cases in Cape Verde [2, 4]. The data contradict the typical profile based on women and children under-five years of age. Official data reports [2, 4] have indicated that more males are affected by malaria, and identified high-risk occupations, including construction or agricultural workers, security guards and the homeless. Only one agricultural worker and 4 security guards appeared in this data, which was dominated by tertiary-sector activities, students and unemployed. The geographical pattern of the samples overlaps with those previous malaria hotspots, including Várzea, Chã de Areia, Fazenda, Achadinha Paiol, and Lém Ferreira neighbourhoods [2, 4]. Further, the results showed that all neighbours and family members tested were negative for malaria (serology and PCR data), which reinforces that infection might be occurring outside of households.

The first WGS for Cape Verdean parasites was performed, and it clearly showed that the analysed samples cluster together and have a very high level of similarity, which suggests that possible the outbreak resulted from clonal expansion of local parasites. The Cape Verdean samples also cluster with isolates from West Africa, as expected. It would be possible to evaluate the robustness of these findings by increasing the number of Cape Verdean parasite samples undergoing WGS, including by considering samples from other years.

### Drug resistance associated molecular markers

Artemether + lumefantrine (AL), is the recommended first treatment line for uncomplicated *P. falciparum* malaria in Cape Verde [6]. Apart from therapeutic efficacy studies (TES), the WHO recommends the use of molecular markers to monitor the emergence of mutations associated with resistance to anti-malarial drugs to detect emerging resistance and prevent potential future treatment failure [29]. Anti-malarial resistance genes polymorphism were assessed by Sanger and Next Generation Sequencing (NGS), the methods were used to screen for mutations in a set of malaria-positive blood samples targeting the *pfcrt*, *pfmdr1*, *pfk13, pfdhps,* and *pfdhfr* genes, which have previously been associated with anti-malarial resistance. The prevalence of the different resistance alleles in Cape Verde was not known, either after or before the introduction of ACT in the country. The prevalence of *pfcrt* (chloroquine resistance marker)-resistant haplotype CVIET remains high, only one of the analysed isolates carried the wild-type haplotype CVMNK. Despite the implementation of ACT in Cape Verde (since 2006) and the withdrawal of chloroquine, the frequency of the resistant allele 76T remains high. Although chloroquine resistant *pfcrt* genotype prevalence follows the trend of continental Africa [30–32], the origin is probably different. In Cape Verde it possibly reflects the limited recombination opportunity, due to the very low malaria transmission over the recent decades. Nevertheless, the identification of the wild-type allele K76 is in line with findings in other malaria regions, where AL is the predominant ACT [33–36]. Probably, due to lumefantrine, regimens tend to select wild-type pfcrt alleles [37–39]. It is generally reported that the mutant haplotype 51I/59R/108N in *pfdhfr* confers resistance to pyrimethamine while 437G/540E in p*fdhps* confers resistance to sulfadoxine. Combined, known as the quintuple mutant, they confer resistance to SP [40–43]. For the *pfdhps* gene the wild-type (S436/A437/K540) haplotype was identified in all analysed samples and for *pfdhfr* the triple mutation S108N/N51I/C59R was detected also in all samples successfully analysed, consistent with other African populations, such as Malawi [22] or Senegal [44]. SP has never been used as first-line treatment for *P. falciparum* uncomplicated malaria in Cape Verde, nor has it ever been used as intermittent preventive treatment (IPT) in infants and pregnant women.

The *pfmdr1* amino acid mutations that have been implicated in multidrug-resistant phenotypes include N86Y, Y184F and D1246Y [45–48], where the NFD haplotype is suspected to be involved in parasite tolerance to AL [49–51]. In this study there was high prevalence of the 184F (89.7%) allele as well as the NFD haplotype (detected in 80% of the clinical isolates analysed). The NFD haplotype has also been identified as the most commonly reported *pfmdr1* haplotype in continental Africa, where AL is widely used as first-line ACT to treat falciparum malaria [11, 47, 52–54]. Regarding the *pfk13,* all samples were identical to 3D7 strain, only one sample carried 2 mutations R645T and E668K, which are not thought to be resistance related. This outcome was expected as the overall prevalence of SNPs in the *pfk13* is reported to have low prevalence outside Southeast Asia and high diversity among African parasites [1, 55]. The 2 SNPs identified in *pfk13* gene in this study do not coincide either with the most reported or with the non-validated SNPs associated with delayed artemisinin parasite clearance in Africa [55, 56]. Although in Africa, artemisinin partial resistance has not yet been confirmed [1], there have been unconvincing reports of treatment failure in travellers returning from Africa [57, 58]. Mutations in *pfk13*, occurring in more than one African region affected by malaria, are increasing [35, 59–65], evidencing the need to close monitor this gene.

The Cape Verdean population has low immunity to malaria, 57% live on Santiago and Boavista islands where local malaria transmission occurs, the rest of the population lives in islands where no transmission occurs or abroad [6, 9]. Prevention measures are essential to reduce the risk of malaria transmission and the potential for malaria epidemics. Cape Verde is a country in the elimination phase of malaria, with indoor residual spraying with insecticides a major control strategy. This is supported by good case management with diagnostic tests and in-patient treatment of all cases [66]. Other actions recommended by the WHO are chemoprophylaxis and the use of mosquito nets. However, insecticide-impregnated mosquito nets are less used in Cape Verde, where a recent study shows that only 19% of the population use them, but if offered them, 91% would use it [4]. There is the potential to reduce the rate of infection, morbidity and mortality, through the very cost-effective mosquito net malaria prevention strategy [67].

## Conclusion

The majority of the parasite samples analysed shared the same polymorphisms in the drug resistance-associated genes. Polymorphisms in the *pfk13* gene associated with ACT tolerance in Southeast Asia were not detected, but the majority of the tested samples carried the *pfmdr1* haplotype NFD. The first WGS for Cape Verdean parasites was performed and showed that all samples cluster together, have a high level of similarity and are close to West African parasite populations.

## Supplementary Information

**Additional file 1.** Number of malaria cases in Praia city per neighbourhood (N).

## Data Availability

The *P. falciparum* genome sequences used in this study are available in Genbank with the following accession numbers: MW712599-MW712662,MW579763-MW579766.
