# Supplementary material for: Drug resistance profile and clonality of Plasmodium falciparum parasites in Cape Verde: the 2017 malaria outbreak
Source: Malar J. 2021 Mar 31;20:172. doi: 10.1186/s12936-021-03708-z (PMC8011132; doi:10.1186/s12936-021-03708-z)
Supplement: Supplementary file 1 — Additional file 1. Number of malaria cases in Praia city per neighbourhood (N). [file 12936_2021_3708_MOESM1_ESM.docx]

**Supplementary Table 1**  **Sociodemographic characteristics, travel and malaria history of cases and close contacts (family and neighbours)**

| **Variables** | **Malaria cases**  **(n=131)** | **Close contacts**  **(n=137)** | ***p*** |
| --- | --- | --- | --- |
| **Age** **in years** (Mean±SD)  Median (Min-Max) | 32.4±15.3  30 (3-86) | 40.3±18.6  39 (1-83) | <0.001 |
| **Gender n (%)** |  |  | <0.001 |
| Male | 86 (65.6) | 37 (27.0) |  |
| Female | 45 (34.4) | 100 (73.0) |  |
| **Nationality n (%)** |  |  | 1.000 |
| Cape Verde | 130 (99.2) | 135 (98.5) |  |
| Other | 1 (0.8) | 2 (1.5) |  |
| **Marital status n (%)** |  |  | <0.001 |
| Single | 96 (73.3) | 66 (48.9) |  |
| Married | 12 (9.2) | 25 (18.5) |  |
| Civil union | 23 (17.6) | 38 (28.1) |  |
| Divorced/widowed | 0 (0.0) | 6(4.4) |  |
| **Educational level n (%)** |  |  | 0.010 |
| Never attended school | 9 (6,9) | 19 (14.1) |  |
| Basic School | 43 (32.8) | 61 (45.2) |  |
| Secondary School | 61 (46.6) | 39 (28.9) |  |
| High school | 9 (6.9) | 8 (8.1) |  |
| Other | 9 (6.9) | 5 (3.7) |  |
| **Professional status n (%)** |  |  | 0.150 |
| Unemployed | 18(13.8) | 18 (13.4) |  |
| Primary sector | 1(0.8) | 0 (0.0) |  |
| Secondary sector | 5(3.8) | 11(8.2) |  |
| Tertiary sector | 74(56.9) | 79(59.0) |  |
| Retired | 1(0.8) | 5(3.7) |  |
| Student | 31(23.8) | 21 (15.7) |  |
| **Travelled in the past six months n (%)** |  |  | 0.060 |
| Yes | 7 (5.6) | 16 (11.8) |  |
| No | 124 (94.7) | 120 (88.2) |  |
| **History of malaria n (%)** |  |  | 1.000 |
| Yes | 2 (1.2) | 3 (2.2) |  |
| No | 129 (98.5) | 133 (97.8) |  |
| **Know a malaria case * n (%)** |  |  | <0.001 |
| Yes | 10 (7.6) | 82 (60.3) |  |
| No | 121 (92.4) | 54 (37.7) |  |
| **Fever in the past year* n (%)** |  |  | 0.027 |
| Yes | 7 (5.4) | 18 (13.3) |  |
| No | 123 (94.6) | 135 (86.7) |  |
| **Signs and symptoms of malaria n (%)** |  |  | NA |
| Fever | 127 (97.7) | - |  |
| Headache | 101 (77.7) | - |  |
| Chills | 84 (64.6) | - |  |
| Vomiting | 35 (26.9) | - |  |
| Body pain | 70 (53.8) | - |  |
| Other | 41 (31.5) | - |  |
| **Length of hospital stay in days** Median (P_25_-P_75_)  (Min-Max) | 4 (3-4)  (3-30) | - |  |

*In the past year

**Supplementary Table 2** **Primers and thermocycling conditions for *Plasmodium* species identification**

| **Genus and species** | **Primers** | **Primer sequence (5’-3’)** | **PCR conditions** |
| --- | --- | --- | --- |
| *Plasmodium* | *rPLU5* | CTTGTTGTTGCCTTAAACTTC | 95ºC for 3 min; 25x [95ºC for 1 min; 58ºC for 1 min; 72ºC for 2min]; 72ºC for 3 min. |
|  | *rPLU6* | TTAAAATTGTTGCAGTTAAAACG |  |
| *P. falciparum* | *rFAL1* | TTAAACTGGTTTGGGAAAACCAAATATATT | 95ºC for 3 min; 35x [95ºC for 1 min; 58ºC for 1 min; 72ºC for 2 min]; 72ºC for 3 min. |
|  | *rFAL2* | ACACAATGAACTCAATCATGACTACCCGTC |  |
| *P. malariae* | *rMAL1* | ATAACATAGTTGTACGTTAAGAATAACCGC |  |
|  | *rMAL2* | AAAATTCCCATGCATAAAAAATTATACAAA |  |
| *P. vivax* | *rVIV1* | CGCTTCTAGCTTAATCCACATAACTGATAC |  |
|  | *rVIV2* | ACTTCCAAGCCGAAGCAAAGAAAGTCCTTA |  |
| *P. ovale* | *rOVA1* | ATCTCTTTTGCTATTTTTTTAGTATTGGAGA |  |
|  | *rOVA2* | GGAAAAGGACACATTAATTGTATCCTAGTG |  |

**Supplementary Table 3** **Primers and thermocycling conditions for PCR amplification of *pfmdr1* and *pfk13* gene fragments**

| Gene | Codon | Primer | Primer (5’-3’)’ | PCR conditions |
| --- | --- | --- | --- | --- |
| *Pfmdr1* | *86/184* | *AF* | ATGGGTAAAGAGCGAAAGAG | 94ºC for 2 min; 10x [94ºC for 1 min; 60ºC for 30 sec; 72ºC for 1 min.]; 72ºC for 3 min. |
|  |  | *AR* | GTCTTTTCTCCACAATAACTTGC |  |
|  |  | *ANF* | GTATGTGCTGTATTATCAGGAGGA |  |
|  |  | *SEQ* | AACAGTTCTTATTCCCATTAAAGCC |  |
|  | *1246* | *1246F* | CTACAGCAATCGTTGGAGAA |  |
|  |  | *1246R* | GAGAATAGCTATAGCTAGAGC |  |
| *Pfk13* |  | *Pfk 13F* | GAAATCCGTTAACTATACCC | 94ºC for 2 min; 10x [94ºC for 1 min; 57ºC for 30 sec; 72ºC for 1 min.]; 72ºC for 3 min. |
|  |  | *Pfk 13R* | GGAGTGACCAAATCTGGGA |  |
|  |  | *Pfk13NF* | GAGATGTATGGTATGTTTCAAG |  |
